# The Discovery of Natural Gifts

**Published:** 1888-06-16

**Authors:** 


### THE DISCOVERY OF NATURAL GIFTS.

WITH boys, education is now a fairly satisfactory process, and efforts are diligently made to find out the inborn tendencies of each. Even in the "good old days," as they are called, some attempt was made to place each son in that position in life for which he seemed best fitted. The brightest youth, for instance, was sent to study law, and the dullest blockhead was destined for the church. But in the case of girls it is now only beginning to be recognised that an attempt should be made to discover their natural gifts, and to pursue an appropriate training in accordance with such endowments. We are beginning to learn that it is worse than useless to waste time over the attempt to learn what are euphemistically termed accomplishments, when there is not the faintest possibility that they can be learned. Many a conscientious and hard-working girl undergoes untold misery at school, and suffers all her life in consequence of failure in her diligent attempts to achieve the impossible

